# Transcriptional Modulation of Intestinal Innate Defense/Inflammation Genes by Preterm Infant Microbiota in a Humanized Gnotobiotic Mouse Model

**DOI:** 10.1371/journal.pone.0124504

**Published:** 2015-04-30

**Authors:** Lei Lu, Yueyue Yu, Yuee Guo, Yunwei Wang, Eugene B. Chang, Erika C. Claud

**Affiliations:** 1 Department of Pediatrics/Neonatology, University of Chicago, Chicago Illinois, United States of America; 2 Department of Medicine/Gastroenterology, University of Chicago, Chicago Illinois, United States of America; 3 Internal Medicine Residency Program at Reno, University of Nevada School of Medicine, Reno Nevada, United States of America; CWRU/UH Digestive Health Institute, UNITED STATES

## Abstract

**Background and Aims:**

It is known that postnatal functional maturation of the small intestine is facilitated by microbial colonization of the gut. Preterm infants exhibit defects in gut maturation, weak innate immunity against intestinal infection and increased susceptibility to inflammatory disorders, all of which may be related to the inappropriate microbial colonization of their immature intestines. The earliest microbes to colonize the preterm infant gut encounter a naïve, immature intestine. Thus this earliest microbiota potentially has the greatest opportunity to fundamentally influence intestinal development and immune function. The aim of this study was to characterize the effect of early microbial colonization on global gene expression in the distal small intestine during postnatal gut development.

**Methods:**

Gnotobiotic mouse models with experimental colonization by early (prior to two weeks of life) intestinal microbiota from preterm human infants were utilized. Microarray analysis was used to assess global gene expression in the intestinal epithelium.

**Results and Conclusion:**

Multiple intestinal genes involved in metabolism, cell cycle regulation, cell-cell or cell-extracellular matrix communication, and immune function are developmental- and intestinal microbiota- regulated. Using a humanized gnotobiotic mouse model, we demonstrate that certain early preterm infant microbiota from prior to 2 weeks of life specifically induce increased NF-κB activation and a phenotype of increased inflammation whereas other preterm microbiota specifically induce decreased NF-κB activation. These fundamental differences correlate with altered clinical outcomes and suggest the existence of optimal early microbial communities to improve health outcomes.

## Introduction

The human gastrointestinal tract is densely colonized with approximately 10^14^ resident bacteria [1] which provide many benefits to the host including supply of essential nutrients, metabolism of indigestible compounds, defense against colonization by pathogens and influence on the development of intestinal architecture [2–8]. In human infants, colonization by microorganisms begins around birth [9]. Early assembly of the gut microbiota has been linked to the terminal differentiation of intestinal structures [10] and the development of innate immune responses under optimal conditions [11,12]. This microbial colonization has also been linked to disease states like necrotizing enterocolitis[13,14].

Studies on fecal samples collected from term and preterm infants suggest that early gut colonization influences infant health and disease, and that microbial interventions may affect disease risk both early and late in life [15,16]. Studies have also consistently shown that the microbiota of hospitalized, preterm infants differs greatly from that of healthy, full-term babies [17–20]. The immaturity of the preterm infant gut and immune system likely leads to the increased sensitivity and responsiveness to gut colonizing bacteria seen when compared to full term controls (reviewed in [21]).

Many disorders of preterm newborns are inflammatory in nature including chronic lung disease[22] and neonatal necrotizing enterocolitis[23]. Furthermore, several early clinical observations suggest a link between factor(s) produced during chronic inflammation and growth failure [24,25]. For this study, we hypothesized that early colonization of the developing preterm gut influences intestinal epithelial function and inflammatory potential.

GeneChip profiling of transcripts in ileal mucosal RNAs was used to assess the effects of microbiota on gene expression in the murine intestine during postnatal development. Three classes of mice of the same C57B6 genetic background but with distinct intestinal microbial phenotypes were used: GF mice (germ free, without microbiota); SPF mice (specific pathogen free, endogenous murine microbiota) or GF-M_PI_ (germ free conventionalized with human preterm infant-PI- microbiota). This experimental design revealed both age-dependent and microbial colonization induced changes in small intestine gene expression. Differences in influence of certain microbial communities on intestinal inflammatory and immune pathways suggest existence of optimal communities to modulate the exaggerated inflammatory response of the immature gut. Our studies present a powerful model for dissecting the function of the microbiota and microbiota/host relationships in ways that would not be possible in human infants. Furthermore, our studies suggest that early colonization patterns in certain preterm infants lead to an exaggerated inflammatory state that may affect growth and susceptibility to the multiple morbidities of inflammatory origin seen in preterm infants.

## Material and Methods

### Subjects

This study was designed to investigate the influence of early microbiota on the developing gut using gnotobiotic mice transplanted with early microbiota from preterm infants. Subjects were recruited from the neonatal intensive care unit (NICU) at The Comer Children’s Hospital of the University of Chicago. As growth can be used as a marker of health in infants, we arbitrarily selected two preterm human infants with normal >10gm/k/day weight gain or decreased <10gm/kg/day weight gain as the donors for microbiota transplantation. The two preterm infants were comparable in gestational age (27-week GA), mode of delivery (cesarean section), feeding pattern (breast fed) and exposure to antibiotics (48 hour administration of ampicillin and gentamicin immediately after birth). Based on our previously published work demonstrating a temporal progression of preterm infant microbiota with distinct clustering at <2 weeks of life[26], the early microbiota (<2 weeks of life) from preterm infants with normal growth (M_PI_-H) and low growth (M_PI_-L) were collected for further analysis and transfaunation studies. At the time the samples were obtained, both were receiving total parenteral nutrition (TPN) intravenously, as well as their own mother’s non-fortified breast milk from frozen stores via nasogastric tube.

### Subject Compliance/Protection and Informed Consent

Specimen collection procedures involved minimal physical risk to subjects. The procedures were approved by the Institutional Review Board (IRB), approval 14991B, and written consent was obtained from patient parents.

### Mice

All procedures were carried out in accordance with Institute guidelines at the University of Chicago. Germ free (GF) C57BL/6J mice were maintained in the gnotobiotic facility of the Digestive Disease Research Core Center (DDRCC) at the University of Chicago. GF colonies are routinely tested for microbes and parasites by the facility’s staff to ensure germ-free conditions. Conventional C57BL6J mice (From the Jackson Laboratory) were bred and housed in the animal care facilities of the University of Chicago under specific pathogen free environment (SPF) conditions. All groups of mice were allowed ad-libitum access to Harlan Teklad 7012 (SPF) or its autoclaved equivalent NIH 31 (GF) chow. All animal studies were approved by the Institutional Animal Care and Use Committee of the University of Chicago (permit number 71703).

### Colonization Experiments

To initiate microbial colonization, pregnant GF female 8- to 9-week-old mice (estimated between E15-17) were gavaged with 0.25 ml of freshly prepared fecal homogenate from frozen fecal samples of the preterm human infant donors (M_PI_-L, M_PI_-H). Pups delivered spontaneously and naturally acquired the microbiota of interest. Litters remained with the mother to allow natural passage of intestinal microbes. These pups were studied in parallel with age-matched SPF and GF controls.

### DNA extraction, 16S rDNA amplification, sequencing analysis and taxonomic classification of cloned rDNA PCR products

DNA was extracted from stool samples using the QIAamp Stool DNA kit (Qiagen) according to manufacturer’s instruction. PCR primers used were specific for the 533–786 base pair (bp) region of the 16S rRNA encoding gene (515F: 5’-GTGCCAGCMGCCGCGGTAA-3’ and 806R: 5’- GGACTACHVGGGTWTCTAAT-3’) and contained Illumina 3' adapter sequences as well as a 12 bp barcode [27]. Sequencing was performed by the Next Generation Sequencing service (Roche 454 GS FLX+ Amplicon Sequencing) at Research and Testing Laboratory (Lubbock, TX). Sequences were trimmed and then analyzed using QIIME[28]. Using the QIIME wrappers, operational taxonomical units (OTUs) were picked at 97% sequence identity using uclust and a representative sequence was then chosen for each OTU by selecting the most abundant sequence in that OTU. These representative sequences were classified and assigned a taxonomic string using the RDP Classifier [29,30]. These datasets were deposited in MG-RAST under the link http://mg-rast.mcs.anl.gov/linkin.cgi?metagenome (accession numbers 4614878.3, 4614879.3, 4614885.3, 4614881.3, 4614880.3, 4614884.3, 4614883.3, 4614877.3, 4614876.3, 4614875.3, 4614874.3, 4614886.3, and 4614882.3).

### Microarray Hybridizations and Data Analysis

Total RNA from small intestinal mucosal scrapings was extracted from pre and postweaned GF (n = 2, same litter) and SPF (n = 2, same litter) using the RNeasy kit and RNase-free DNase I (QIAGEN) digestion based on the manufacturer's protocol. RNA integrity was determined using an Agilent 2100 Bioanalyzer (Agilent Technologies). Microarray was done by Onearray (Phalanx, Belmont, CA). Six micrograms of total RNA was reverse transcribed to cDNA by Ambion Amplification then transcribed into biotin-labeled cRNA with the One-Cycle Target Labeling Kit (Phalanx)). Hybridization was done in triplicate to the Mouse Array (mouse v2), and chips were scanned with a Molecular Devices GenePix Scanner and Image quality analysis performed with Rosetta Resolver. The database has been submitted to the GEO repository (http://www.ncbi.nlm.nih.gov/geo, *accession code*: *GSE44788*).

Microarray analysis was performed using Array Studio software from Omicsoft. The data were normalized with gcRMA and subsequently adjusted for multiple testing by Benjamini and Hochberg false discovery method (p<0.05). For in-depth functional analysis of the microarray data, all differentially expressed genes (p<0.05) were imported into MetaCore analytical software (GeneGo) to generate gene enrichment reports based on *p* values calculated with hypergeometric distribution.

### Quantitation of Gene Expression with Real-Time PCR

RNAs were isolated from ileal tissues as described above. qRT-PCR was performed using mouse-specific primers with SYBR-Green PCR master mix (Applied Biosystems). For all the genes tested, the primer sets were selected and obtained from the Harvard primer Bank (http://pga.mgh.harvard.edu/primerbank) [31]. cDNA samples were assayed in duplicate and gene expression levels for each sample were normalized relative to GAPDH with delta-deltaCt (ΔΔCt) calculation.

### Cytokine Assay

Sera from 3 wk old M_PI_, SPF and GF mice was collected and inflammatory cytokines were measured by BioPlex assay (BioRad) according to manufacturer's instructions.

### Histology and immunohistochemistry

Distal ileum segments were fixed in formalin and paraffin embedded for sectioning and histological examination. For immunohistochemistry, 5-μm paraffin sections were dehydrated and endogenous peroxidase was quenched with H_2_O_2_ (3% in methanol). Antigen retrieval was performed by microwave in a citrate buffer and endogenous avidin-biotin blocked (Vector Laboratories, Burlingame, CA). Primary antibodies or isotype control were added to the sections, followed by biotinylated secondary antibody and ABC detection kit (Vector Laboratories). Sections were counterstained with hematoxylin (DAKO, Carpinteria, CA) and examined on a BioRad Axiophot fluorescence photomicroscope.

### Statistics

The ΔΔ’ (CTs) were used to calculate the expression levels of amplified genes and were normalized to expression levels of GAPDH. The nonparametric Kruskal-Wallis test was used for comparing ΔΔ’ (CTs) between M_PI_-L and M_PI_-H. Differences were considered significant when *p*<0.05. ANOVA analysis and a post-hoc Tukey honest significance (HSD) test were used to compare differences among groups. Findings are presented as the mean ± standard error and a *p*<0.05 was considered statistically significant.

## Results

### Identification and functional classification of age-dependent gene expression in the small intestinal mucosa

We hypothesized that temporal development and bacterial colonization affect small intestinal gene regulation in early life. First, a genome-wide expression analysis was used to examine the patterns of gene expression profiles and to identify functional networks corresponding to temporal development (preweaned vs. postweaned). To separate developmental changes from alterations due to microbial colonization, postweaned vs preweaned was examined in both GF and SPF mice. Out of a total 14626 genes, 4228 genes in GF and 2782 genes in SPF mice were differentially expressed with at least two-fold change between pre and postweaned (2 weeks vs 4 weeks) ileum, (p<0.05). We have termed the difference between pre and post weaned the developmental difference.

**Fig 1:**
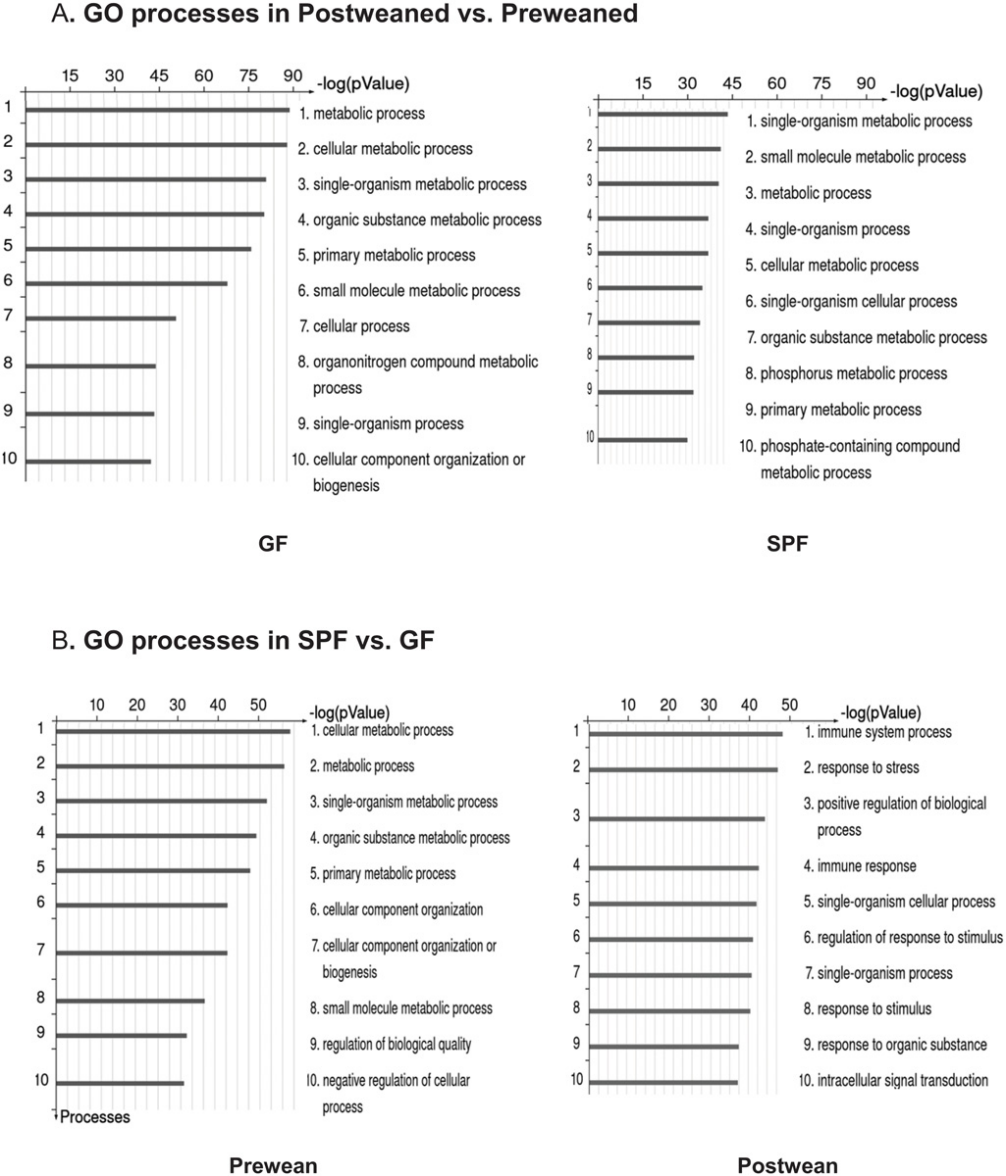
MetaCore gene enrichment analysis of ileum microarray data representing development- and microbial colonization- effects in GF and SPF mice. Differentially expressed genes (fold change ≥ ±2) in SPF and GF ileum from preweaned (n = 2) and postweaned (n = 2) mice were subjected to gene enrichment analysis using GeneGo MetaCore software. Panel A: Development dependent genes; top 10 overrepresented gene ontology (GO) biological processes in GF and SPF ileum (post vs preweaned). Panel B: Microbiota dependent genes; top 10 overrepresented gene ontology (GO) biological processes in pre and postweaned ileum (SPF vs. GF).

The age-dependent differentially expressed genes (preweaned vs postweaned) were subjected to integrated pathway enrichment analysis using MetaCore knowledge-based annotation to extract functional gene expression patterns in GF or SPF ileum. Ranking of relevant integrated pathways was based on hypergeometric *p* values. The top scored Gene ontology (GO) processes overrepresented by differentially expressed gene sets are shown in Fig 1. The differentially expressed genes were predominately associated with metabolism, as the top three scored GO categories were metabolic process, cellular metabolic, and single-organism metabolic process in GF; single-organism metabolic, small molecule metabolic and metabolic process in SPF mouse respectively (Fig 1A).

### Identification and functional classification of microbiota induced gene expression in the small intestinal mucosa

Next, to examine the role of gut microbiota on gene expression pattern separate from developmental changes, we compared the pre or postweaned ileal mRNA expression profiles of SPF to that of GF mice. Gut microbiota colonization had a greater effect in preweaned ileum (preweaned SPF vs. preweaned GF) than in postweaned ileum (postweaned SPF vs. postweaned GF) with 4516 vs 2825 genes affected respectively. Our data indicate that gut microbiota induce large effects on metabolic processes, phosphorylation, organ (heart) development and apoptosis in preweaned SPF vs preweaned GF ileum, whereas the microbiota invoke a strong effect on immune system process, response to stress, positive regulation of biological process and immune response in postweaned SPF vs postweaned GF ileum (Fig 1B).

A large number of differentially expressed genes (1207, fold change ≥± 2) were shared between GF- and SPF-dev (preweaned vs. postweaned) suggesting that these genes were development-associated and not influenced by microbiota colonization. Similarly, we identified 1206 microbiota-associated differentially expressed genes (≥± 2) shared by preweaned and postweaned SPF vs GF, suggesting that these were mainly influenced by microbiota irrespective of developmental stage. We further analyzed these flagged genes in GO biological processes networks. Using GOMiner software in combination with Venn Master[32,33], we identified the top scored overrepresented and overlapping associated networks in the development-associated and microbiota-associated gene sets. These are presented as a Euler diagram (generated using VennMaster) in S1 Fig The development associated genes are primarily involved in the diverse biological functions of protein homooligomeration, mitochondria organization and metabolic processes including lipid, amino acid and vitamin metabolism. The microbiota associated genes preferentially affect biological functions involved in immune and cytokine signaling processes in the host reflected by the overrepresentation of genes involved in IκB and NF-κB biological functions.

### Human preterm infant microbiota transfaunated to mouse pups

Growth of the preterm infant is a surrogate for health [34]. To investigate the effect of microbiota clinically relevant to the preterm infant on the developing gut, we identified two preterm infants with different growth rates as donors of early human fecal samples for this study. Fecal samples from prior to 2 weeks of life from these two infants were analyzed. The sample from the infant with low growth is indicated as M_PI_-L and the sample from the infant with normal growth is indicated as M_PI_-H. The composition of the microbial community in the fecal samples obtained from these preterm infants was analyzed and the frequencies of sequences from each subject were grouped according to public database NCBI and RDP-II. Table 1 lists the all species present at >0.5% of the community.

**Table 1:** The composition of microbiota in M_PI_-L and M_PI_-H.

M_PI_-L_Sequencing ID	% of Total flora	M_PI_-H_Sequencing ID	% of Total flora
Comamonas sp	***18***	Comamonas sp	***21***
Weissella confuse	***14***	Weissella confuse	***13***
Leuconostoc citreum	***14***	Leuconostoc citreum	***10***
Lactococcus sp	***12***	Comamonas testosteroni	***10***
Comamonas testosteroni	***7***	Lactococcus sp	***10***
Citrobacter sp	***5***	Acinetobacter sp	***3***
Delftia sp	***4***	Citrobacter sp	***3***
Lactococcus lactis	***3***	Lactococcus lactis	***3***
Acinetobacter sp	***3***	Delftia sp	***2***
Streptococcus sp	***2***	***Polaromonas sp***	***2***
***Enterobacter sp***	***1***	***Alcaligenes sp***	***2***
Lactococcus raffinolactis	***1***	***Enterococcus italicus***	***1***
***Leuconostoc sp***	***1***	Streptococcus sp	***1***
***Flavobacterium sp***	***1***	***Chryseobacterium bovis***	***1***
***Stenotrophomonas maltophilia***	***0*.*5***	Lactococcus raffinolactis	***1***
***Ralstonia sp***	***0*.*5***	***Aeromonas sp***	***1***
***Moraxella osloensis***	***0*.*5***	***Arcobacter butzleri***	***0*.*5***
Veillonella sp	***0*.*5***	***Brevundimonas sp***	***0*.*5***
***Pseudomonas sp***	***0*.*5***	***Fusobacterium sp***	***0*.*5***
***Acidovorax sp***	***0*.*5***	***Bacteroides sp***	***0*.*5***
***Achromobacter sp***	***0*.*5***	Veillonella sp	***0*.*5***
Weissella cibaria	***0*.*5***	Weissella cibaria	***0*.*5***
***Kordia sp***	***0*.*5***		

The unique bacterial species are in Italic.

Consistent with what has been shown in other analysis of preterm infant fecal microbiota [35], these two preterm infants’ microbiota both exhibited a limited phylogenetic diversity dominated by *Proteobacteria* and *Firmicutes* with a small portion of *Bacteroidetes* and *Actinobacteria*.

**Fig 2:**
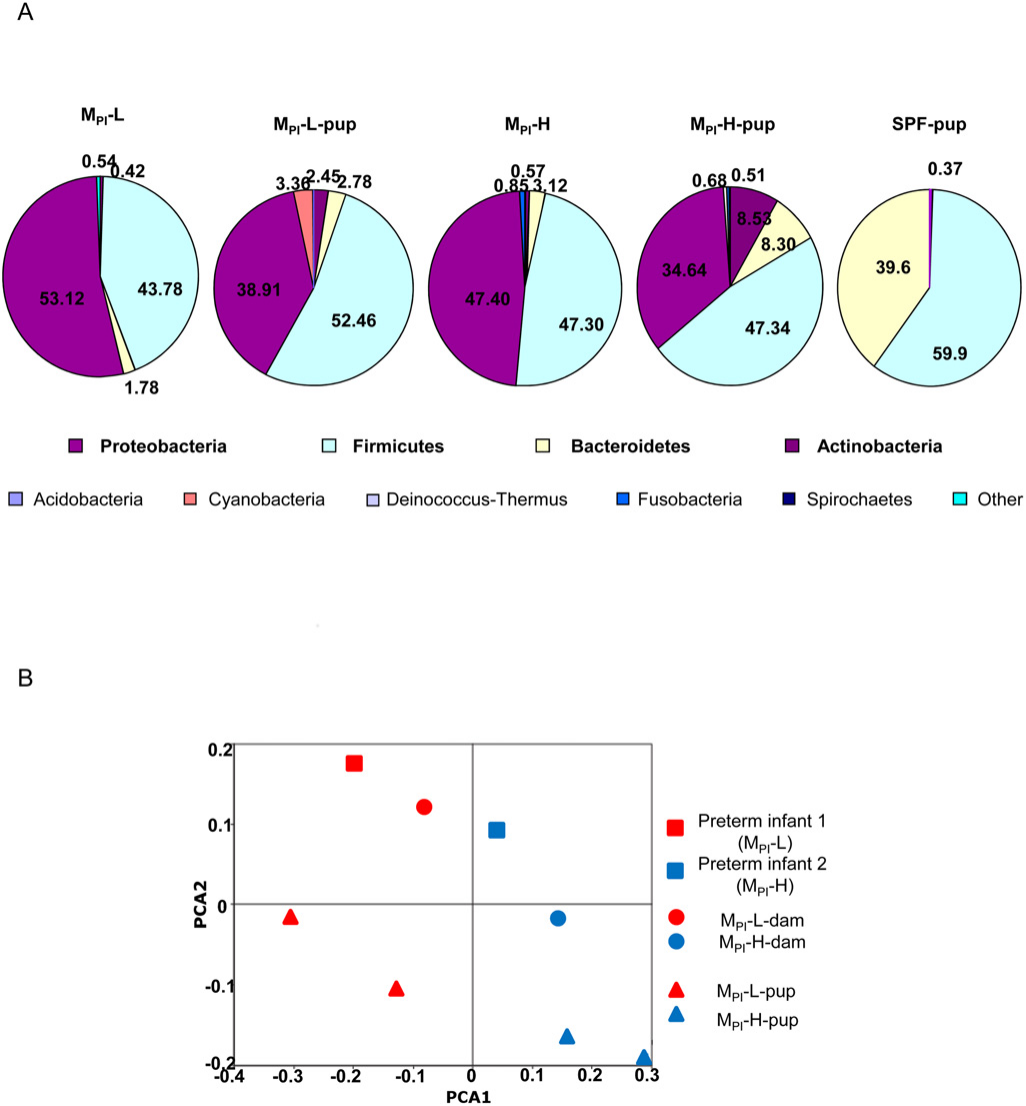
Comparison of bacterial communities following transfaunation of germ free mice with M_PI_ fecal lysates. A: The relative abundance of different bacterial phyla in input M_**PI**_-L,-H fecal samples and from M_PI_-L, H and SPF preweaned mouse pups is shown. B: PCoA analyses of the M_PI_-L and M_PI_-H in original human infants, transfaunated dams and respective pups based on Bray Curtis dissimilarities among samples given presence/absence of major taxa present in at least one sample. Axis 1 explained 27.5% of variation and axis 2, 18.1%.

To best mimic the normal gut microbiota acquisition process, pregnant GF mice were transplanted with early microbiota from the preterm human infants (M_PI_) to facilitate colonization from birth of the resulting pups with the microbiota of interest. Fecal samples from M_PI_ and SPF pups were sequenced and grouped according to their taxonomic group (Fig 2A). Compared to native murine SPF microbiota that is dominated by *Firmicutes* (59.9% relative abundance) and *Bacteroidetes* (39.6%), M_PI_-colonized mouse pups exhibited a very different composition reflecting the input human preterm infant microbiota dominated by *Proteobacteria* and *Firmicutes* in both M_PI_-L and-H colonized mice. There is a greater contribution of *Bacteroidetes* (8.30 vs. 3.42) and *Actinobacteria* (8.53 vs. 0.57) in M_PI_-H-colonized mice compared to M_PI_-L mice. Principal Coordinates Analysis based on Bray Curtis Dissimilarities revealed clear separation of M_PI_-L (red) from M_PI_-H (blue) by grouping into one side of the PCA plot (Fig 2B).

### Early human microbiota affects small intestine gene expression in gnotobiotic mice

**Fig 3:**
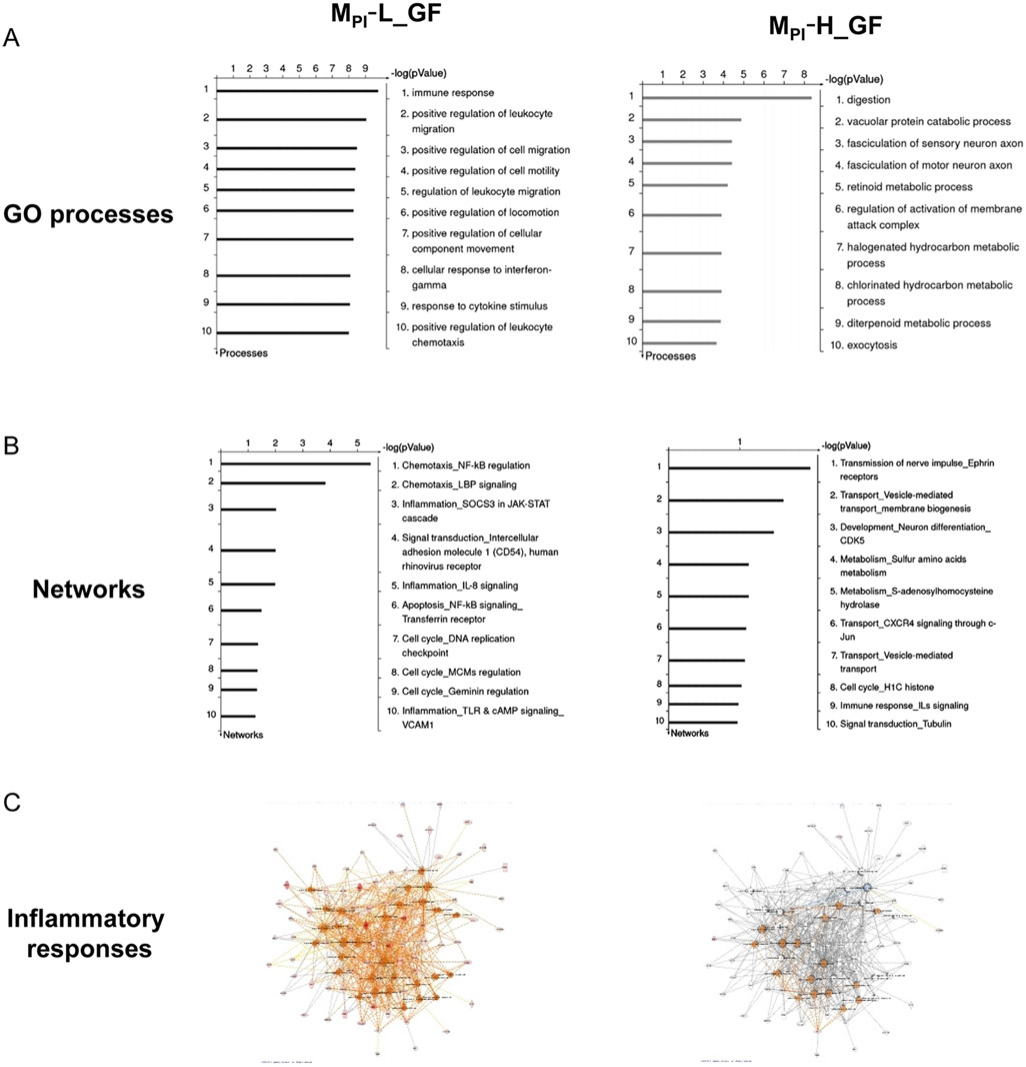
MetaCore gene enrichment analysis of ileum microarray data representing microbiota effects in preweaned M_PI_-L and M_PI_-H-gnotobiotic mice. M_**PI**_-L and M_**PI**_-H induced differentially expressed genes were analyzed by GeneGO MetaCore software. Panel A: Top 10 overrepresented GO biological processes in preweaned M_PI_-L, H vs GF mice. Panel B: Top 10 scored overrepresented networks in preweaned M_PI_-L, H vs GF mice. Panel C: Comparison of the differentially expressed genes in M_PI_L_GF and M_PI_-H_GF presented in inflammatory response-related networks using the IPA program. Orange line: activation; Blue line: inhibition; Grey: not present.

To gain insights into effects of preterm infant microbiota, we collected ileal RNA from preweaned mouse pups transfaunated with preterm infant microbiota for a genome-wide analysis of gene expression profiles. Results were compared to expression data from preweaned GF mouse pups. This comparison yielded a total of 461 differentially expressed genes in which transcript levels had at least twofold change (452 up and 9 down) in M_PI_-L and a total of 842 differentially expressed genes (782 up and 60 down) in M_PI_-H mouse pups. The differentially expressed genes in M_PI_-L and M_PI_-H were further analyzed using GeneGo MetaCore Gene Enrichment analysis. The top scored GO processes and networks overrepresented by differentially expressed genes are presented in Fig 3. When comparing M_PI_ with GF mouse ileum, M_PI_-L affected primarily innate immune/inflammatory responses with many genes involved in leukocyte adhesion, migration and chemotaxis. In contrast, M_PI_-H had little or no effect on these processes.

145 genes in M_PI_-L and 154 genes in M_PI_-H overlapped with the microbiota-associated genes identified in the SPF vs GF analysis. These genes were further analyzed using Ingenuity Pathway Analysis (IPA) which confirmed the findings that M_PI_-L-upregulated genes in inflammatory response networks in general and leukocyte adhesion, migration and chemotaxis in particular (Fig 3C, larger format in S2 Fig). These networks were significantly downregulated or non-represented in the M_PI_-H—induced gene set (Fig 3C). These data suggest microbiota-dependent gene expression changes in the developing host, with certain preterm infant microbiota as represented by M_PI_-L potentially inducing specific strong influences on inflammatory responses.

### Real time PCR for Validation of gene expression microarray findings

**Fig 4:**
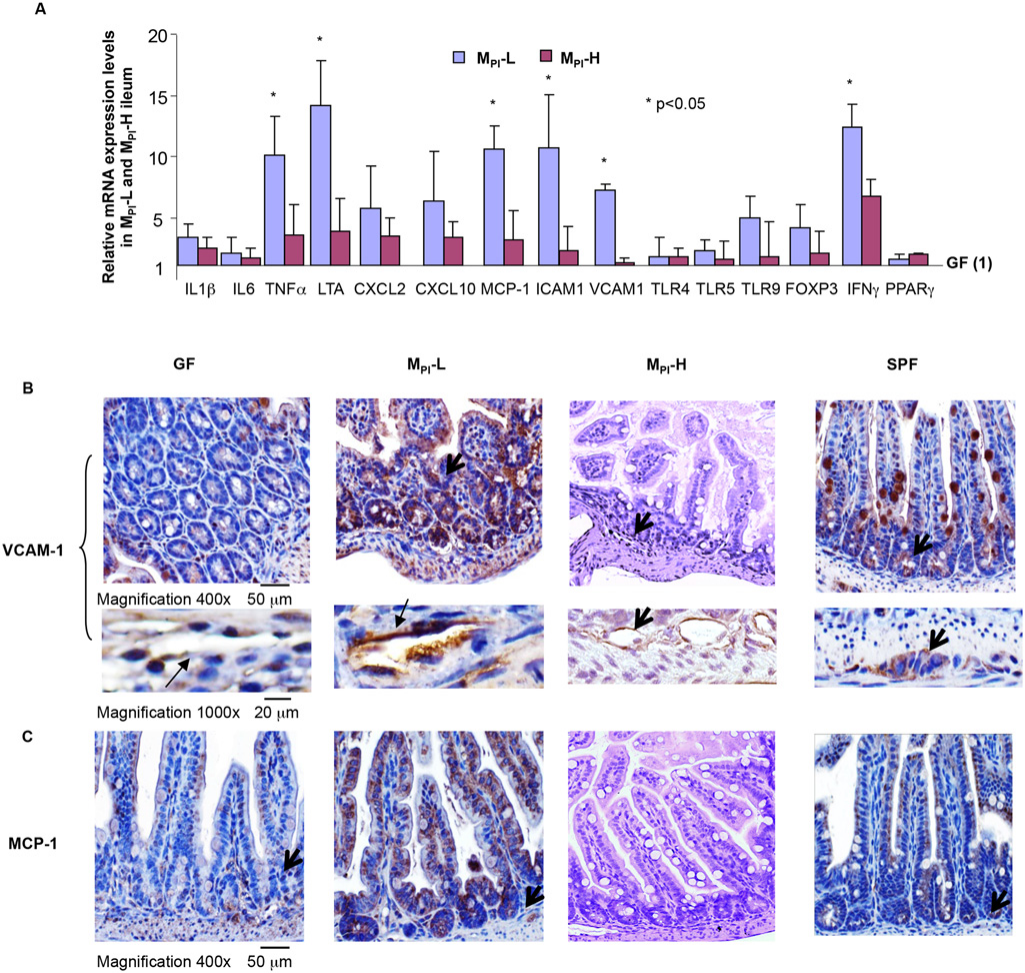
Differential expression of innate immune/inflammatory response-related genes in M_PI_-L,-H by qRT-PCR and immunohistochemistry. A: Ileum RNAs were extracted from preweaned GF (n = 3), M_PI_-L (n = 5) and M_PI_-H (n = 5) gnotobiotic mice. M_**PI**_-dependent gene expression levels were normalized to GAPDH and expressed as fold change over GF (designated as 1). B: The NF-κB-target gene VCAM1 was analyzed by immunohistochemical staining of 5 micron paraffin-embedded distal ileum tissue from preweaned GF, M_PI_-L,-H-gnotobiotic and SPF mice (n = 3/per group) using anti- VCAM1 respectively. Representative areas are shown (magnification 400x), black arrows indicate VCAM-1 expression in ileal epithelium and endothelial cells. C: MCP-1 expression was analyzed by immunohistochemical staining of 5 micron paraffin-embedded distal ileum tissue from 3wk-old GF,M_PI_-L,-H and SPF mice (n = 3/per group) using anti- MCP-1 respectively. Representative areas (n = 3/per group) are shown (magnification 400x), black arrows indicate MCP-1 expression in ileal epithelium.

We next selected inflammation-associated genes that were differentially expressed by gene expression array analysis in M_PI_-L (Fig 3) for further validation by semiquantitative (realtime) RT-PCR and comparison to M_PI_-H. The set included ICAM1, CXCL1, CXCL2, CXCL10, VCAM1, TLR4, 5 and 9, INFγ, FoxP3, IL1βIL6 and TNFα. The average change in mRNA expression of these genes in M_PI_-L and M_PI_-H pups (n = 5 each group) is shown in Fig 4A. Consistent with the expression array data, there was a significant upregulation of inflammatory cytokines (TNFα, IL1β), chemokines (CCL10, MCP-1(CCL2)) as well as adhesion molecules (ICAM-1, VCAM-1) in M_PI_-L compared to that in M_PI_-H mouse ileum tissues.

We next examined VCAM-1 and MCP-1 expression in the ileum tissue of GF, M_PI_s and SPF mice by immunohistochemistry staining. VCAM-1 and MCP-1 are two NF-κB target-genes whose expression levels were upregulated in M_PI_-L based on gene expression array and qPCR. Moreover, they are important molecules implicated in many inflammatory states [36–39]. There were elevated levels of VCAM-1 and MCP-1 in M_PI_-L ileum compared to GF and SPF ileum tissues, while M_PI_-H ileum showed a qualitative decrease in the levels of VCAM-1 and MCP-1 expression compared to M_PI_-L and SPF (Fig 4B and 4C).

### Measurement of serum cytokines as an indication of the systemic inflammatory response

**Fig 5:**
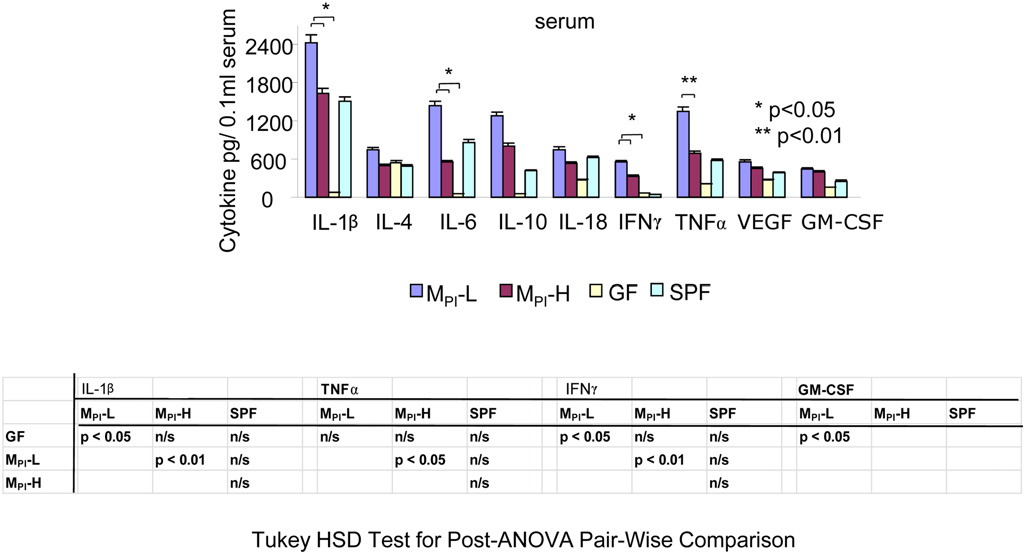
M_PI_ effects on systemic immune and inflammatory cytokine expressions by multiplex immunoassay. Multiplex immunoassay of immune and inflammatory cytokine expressions in serum collected from preweaned GF and M_PI_-L, H and SPF. The levels of these cytokines are presented as mean ± SEM. One-way ANOVA with post-hoc Tukey’s honest significance (HSD) test was used to compare the groups.

Using a multiplex immunoassay we next examined immune and inflammatory cytokine expressions in blood sera collected from GF, M_PI_-L, -H pups (n = 4) at weaning. As shown in Fig 5, one-way ANOVA with post-hoc Tukey’s HSD test showed that the inflammatory cytokine levels (IL1β, TNFαand IFNγ) were significantly elevated in the serum of M_PI_-L compared to M_PI_-H mice. These data suggest an elevated basal systemic inflammatory response in M_PI_-L mice.

### Colonization of M_PI_ induced NF-κB activation in humanized gnotobiotic mouse ileum

**Fig 6:**
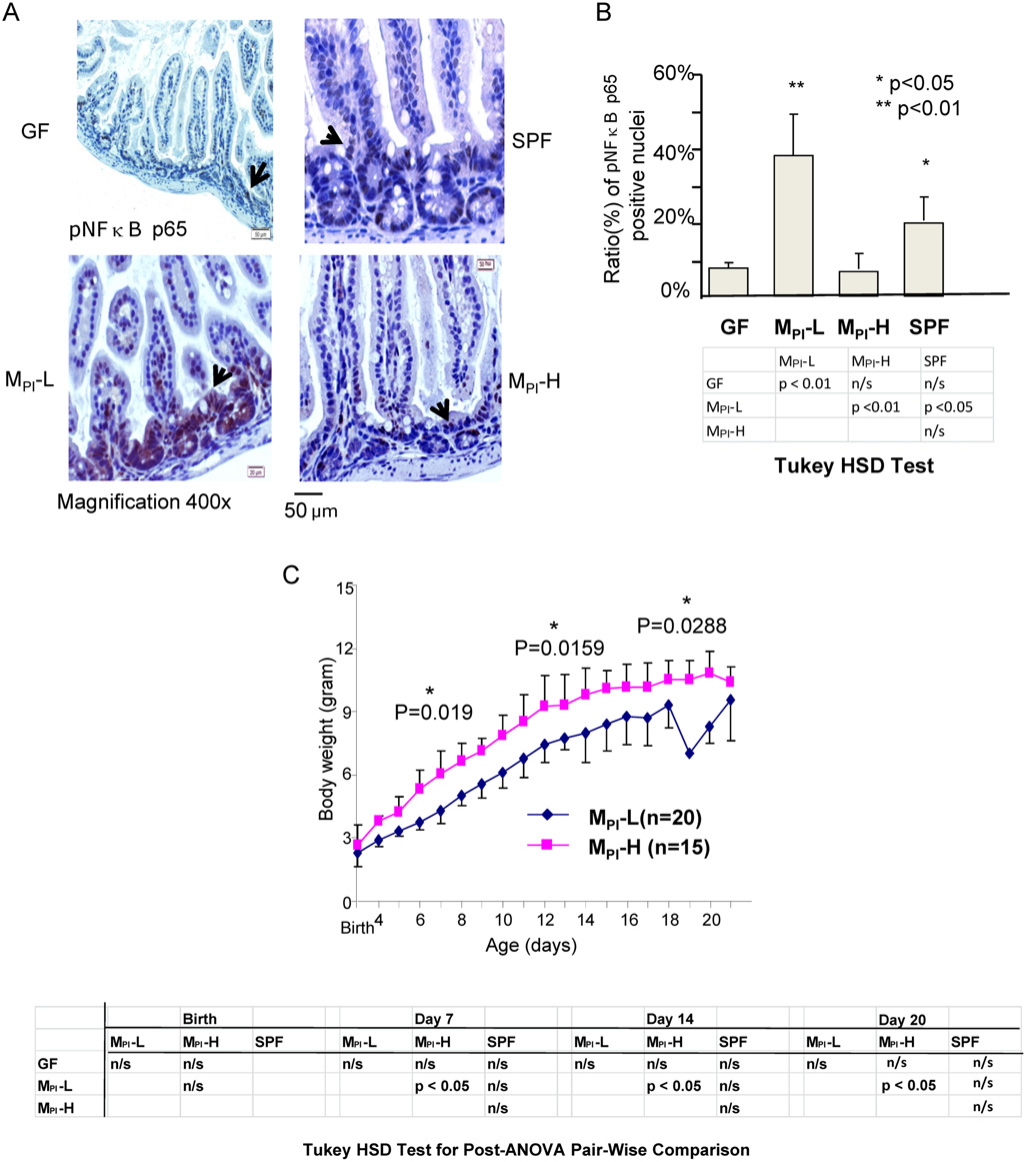
NF-κB activation in M_PI_-L, -H and SPF GF ileum mucosa by immunohistochemistry. A: NF-κB activation was analyzed by immunohistochemical staining of 5 micron paraffin-embedded distal ileum tissue from GF, M_PI_-L, H and SPF mouse. Representative areas (n = 3/per group) are shown (magnification 400x), black arrows indicate nuclear translocation of the phosphorylated NF-κB p65 subunit. B: Nuclear translocation of pp65 was quantified by ImmunoRatio tool from Image J software and presented as % of pp65 positive nuclei/total nuclei. C: Growth curve of M_PI_-L (n = 20) and M_PI_-H (n = 15) from birth to weaning. One-way ANOVA with post-hoc Tukey’s HSD test was used to compare the groups.

To determine if the change in VCAM-1 and MCP-1 expression resulted from *in vivo* evidence of persistent activation of NF-κB in the ileum of M_PI_-L, -H mice, immunohistochemistry analysis was performed to examine baseline nuclear localization of phosphorylated p65 as an indicator of NF-κB activation. The number of phosphorylated p65 positive nuclei in the tissue was quantified using the ImmunoRatio tool from Image J software. As shown in Fig 6A and 6B, preweaned M_PI_-L mouse ileum displayed an increased level of phospho-p65 indicating increased NF-κB activation as compared to GF and SPF mice, while M_PI_-H showed decreased NF-κB activation as compared SPF mice by ImmunoRatio counting. Tukey’s HSD test indicated a significant increase of NF-κB nuclear translocation in M_PI_-L compared to GF, SPF and M_PI_-H mouse ileum (Fig 6B).

To investigate the potential clinical relevance of this difference in baseline inflammation state, we compared the difference in weight gain from birth to 3 week of age. M_PI_-L which was associated with increased NF-κB activation and inflammatory cytokine expression was associated with lower growth, whereas M_PI_-H which was associated with decreased NF-κB activation and cytokine expression demonstrated 20% greater weight gain by weaning (Fig 6C). A one-way ANOVA with post-hoc Tukey’s HSD test demonstrated a significant difference in growth rate between M_PI_-L and M_PI_-H mice at 1, 2 and 3 week of age. This difference in mouse growth phenotype matches the difference in growth phenotype of the preterm infants from whom the fecal sample microbiota transfaunated into these mice originated.

## Discussion and Conclusions

Combining gnotobiotic mouse models with functional genomics, our study is the first to document the wide range of host functions that are modulated after birth by either developmental programming or by the influence of the microbiota on the developing gut. The global gene expression profile changes generated by the cDNA arrays provide a picture of the behavior of gene transcripts in the developing small intestine in GF, SPF and M_PI_ mice that reflects age-dependent and microbial colonization-dependent processes. Our results specifically show that microbiota influence expression of genes important for immune and cytokine signaling processes associated with IκB and NF-κB biological functions.

Studies from our group and others demonstrate that the composition of preterm infant microbiota is very different from full term infant microbiota, and more importantly that immature intestines have very different responses to microbiota compared to that of full term infants [26,29,40,41]. However, the function of microbiota associated with early neonatal gut colonization and its impact on intestinal functions short and long term, can be investigated in much more detail using animal models. Establishing human gut microbiota transplants in gnotobiotic mice is a useful model for translating information about host-microbial mutualism from humans to mice. Our approach was to investigate the effect of early (<2 weeks of life) preterm human infant microbiota as representative of the first microbiota to encounter the naïve developing infant gut, and which thus has the greatest potential to prime key pathways. Gnotobiotic pregnant dams were gavaged with the infant microbiota of interest. Resulting pups acquired the microbiota of interest naturally through birth and nursing in order to recapitulate this clinically relevant progression.

We have previously demonstrated that immature enterocytes have an exaggerated inflammatory response to both commensal and pathogenic intestinal bacteria associated with upregulation of NF-κB signaling [41]. Furthermore, evidence indicates that inflammation inhibits linear growth [42]. Over-expression of high levels of circulating IL-6 caused a growth defect in a murine model (NSE/hIL-6 mice) of inflammation and provided evidence of a direct link between chronic inflammation and stunted growth [43,44]. In this study, we specifically chose early human fecal samples from two preterm infants of the same gestational age and yet with persistent differences in their growth rate from birth until discharge, to further examine the effects of the microbiota on inflammatory responses in developing hosts.

Our data suggest that the interaction between the early microbial community and the preterm gut in its most naïve and immature state may significantly influence intestinal inflammatory states. M_PI_-L (from the host with poor growth) induced a significant upregulation of genes and pathways associated with innate immune/inflammatory responses, particularly in chemotaxis, chemokine/ cytokine signaling, chemokine receptor expression and phagocyte-, monocyte-, and leukocyte-rolling and adhesion in the transfaunated pups. Many of these genes are targets (either direct or indirect) of the NF-κB complex and the changes in their expression levels were associated with increased NF-κB activation (Figs 5 and 6). Since these genes and pathways contribute to host innate protection or inflammation, their heightened expression and constitutive activation in association with certain microbiota may explain the increased propensity to diseases of an inflammatory nature in some preterm infants. In contrast, despite a similar gut microbiota at the phylum level M_PI_-H displayed a downregulation of these inflammation associated genes even compared to SPF colonized mice, suggesting the existence of a microbial community which may protect certain preterm infants.

Unlike full-term infants, preterm infants are still undergoing significant development ex-utero. This development would normally occur in utero without the influence of microbiota. The microbiota with which the immature preterm gut initially comes in contact can significantly influence development. It is potentially a key developmental goal to down-regulate inflammatory responses to colonizing bacteria. The immature preterm gut may not undergo this appropriate response when initially colonized. We found that certain microbiota were associated with a difference in growth in both the mouse pups and human infants from whom the microbiota were obtained. This decrease in growth may be a marker of overall well-being. We realize that it is difficult to make causal association between microbiota and growth based on our small sample size (two preterm infant fecal samples). However, the growth phenotype we observed in human infants was transferred to GF mouse pups via our fecal transfer model. Furthermore, the transfaunated mice reproduced their original hosts’ growth patterns throughout the study. Thus, our data suggest a potential link between improved growth present in M_PI_-H colonized gnotobiotic mouse pups and the decreased or lack of inflammatory responses in these hosts. Furthermore, these data strongly indicate that constitutively elevated NF-κB activation is in part, a molecular mechanism by which early human microbiota influence an inflammatory state.

Importantly these differences in inflammation phenotype, including elevation of systemic inflammatory cytokines, occurred under basal conditions without any secondary inflammatory stimulus. We thus speculate that the different initial microbiota may influence initial inflammatory basal states that could influence susceptibility to inflammatory morbidities of prematurity. The mechanisms by which the microbiota alter the inflammatory phenotype as well as the factors that influence microbiota characteristics in different patients are important next steps for future investigation. The gene enrichment analysis in Fig 1 indicated an influence of microbiota on apoptotic pathways. It is possible that increased apoptosis might lead to the systemic inflammatory response seen in M_PI_-L mice. Importantly these results suggest that certain microbial communities may decrease inflammation and thus protect infants from inflammatory based morbidity of prematurity.

In this study, growth was used as a marker of overall well-being or health. The two patients selected for this study were not intended to link a specific microbial community with a specific clinical outcome. Rather these studies support our main conclusions: 1. Microbiota from a human preterm infant can be transfaunated to a germ free mouse and transfer a phenotype representative of that infant and thus represents a powerful model for studying microbiota function. 2. Early microbiota influence inflammatory potential of the intestinal epithelium. As inflammation is the root of much of the morbidity of prematurity, these studies demonstrate the potential for optimal initial colonization to influence fundamental processes to improve multiple health outcomes. Future investigation will focus on understanding the mechanisms by which early microbiota influence the priming of immune and inflammatory responses of the developing preterm gut. Our goal is to define the causal association between early microbiota and health outcomes of preterm infants. Defining this optimal colonization may enable interventions to influence this earliest microbial colonization to down-regulate inflammatory responses and minimize morbidities of prematurity.

## Supporting Information

S1 Fig Euler diagrams for functional GO analysis of ileum microarray representing development- and microbial colonization- effects in GF and SPF mice. **S1A:** Area-proportional **Euler diagram of** development-associated-genes generated from differentially expressed genes in post vs preweaned GF and SPF ileum (minimum total number of genes per category: 100; max p-value: 0.025) is shown. A total of 10 ontology categories were significantly enriched among the changed genes. The analysis revealed that those categories strongly overlap with metabolic process-related categories. **S1B:** Area-proportional **Euler diagram of** microbial-colonization-associated-genes generated from differentially expressed genes in SPF vs GF ileum. A total of 8 ontology categories were significantly enriched. The analysis revealed that the categories strongly overlap into 2 major categories: cytokine biosynthesis and innate immune response-NF-κB signaling.
(TIF)

S2 Fig Larger format of Fig 3C. Overrepresentation of inflammatory response-related networks in common differentially expressed microbial-associated-genes in M_PI_L_GF (145 genes) vs M_PI_-H_GF (154 genes) by Ingenuity Pathway Analysis (IPA). Orange line: activation; Blue line: inhibition; Grey: not present.
(TIF)

S3 Fig Fig 6C with additional weight information for GF and SPF. Growth curve of M_PI_-L (n = 20), M_PI_-H (n = 15), GF (n = 9) and SPF (n = 9) from birth to weaning. The daily weights are presented as mean (g) ± SEM. One-way ANOVA with post-hoc Tukey’s HSD test was used to compare the groups.
(TIF)
