# Supplementary figures and images for: Transcriptional Modulation of Intestinal Innate Defense/Inflammation Genes by Preterm Infant Microbiota in a Humanized Gnotobiotic Mouse Model
Source: PLoS One. 2015 Apr 30;10(4):e0124504. doi: 10.1371/journal.pone.0124504 (PMC4415773; doi:10.1371/journal.pone.0124504)

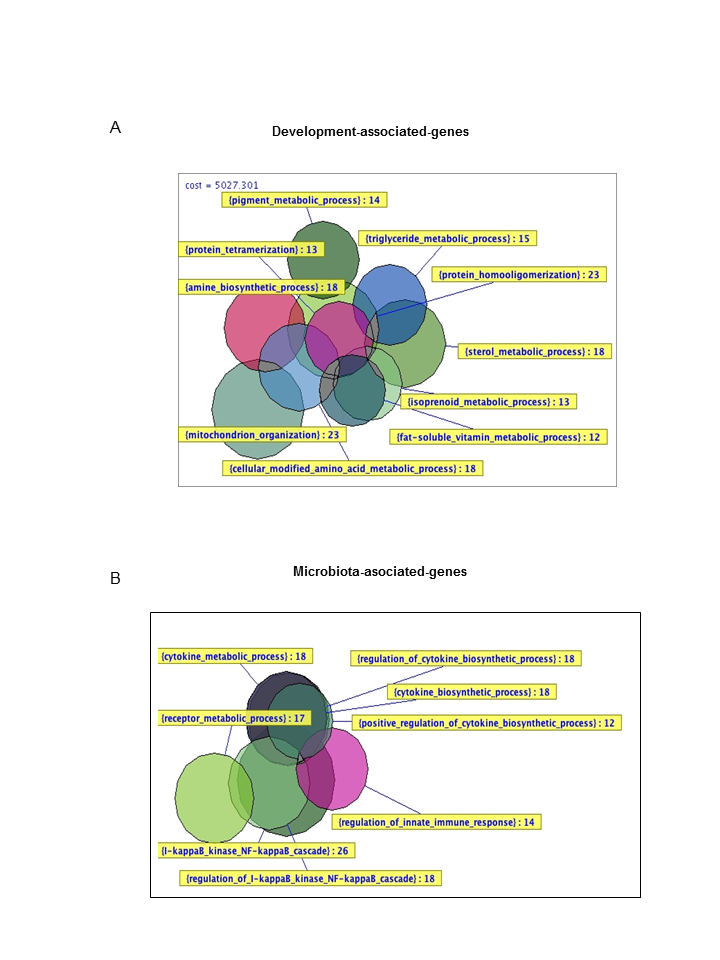

Supplement: S1 Fig — S1A: Area-proportional Euler diagram of development-associated-genes generated from differentially expressed genes in post vs preweaned GF and SPF ileum (minimum total number of genes per category: 100; max p-value: 0.025) is shown. A total of 10 ontology categories were significantly enriched among the changed genes. The analysis revealed that those categories strongly overlap with metabolic process-related categories. S1B: Area-proportional Euler diagram of microbial-colonization-associated-genes generated from differentially expressed genes in SPF vs GF ileum. A total of 8 ontology categories were significantly enriched. The analysis revealed that the categories strongly overlap into 2 major categories: cytokine biosynthesis and innate immune response-NF-κB signaling. (TIF) [file pone.0124504.s001.tif]

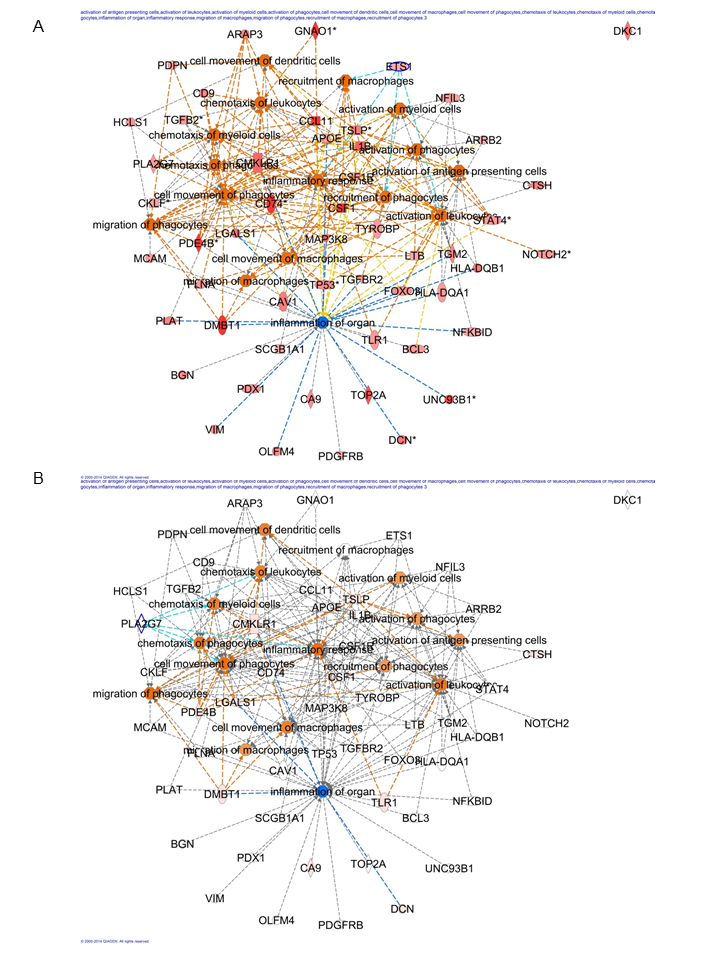

Supplement: S2 Fig — Overrepresentation of inflammatory response-related networks in common differentially expressed microbial-associated-genes in MPIL_GF (145 genes) vs MPI-H_GF (154 genes) by Ingenuity Pathway Analysis (IPA). Orange line: activation; Blue line: inhibition; Grey: not present. (TIF) [file pone.0124504.s002.tif]

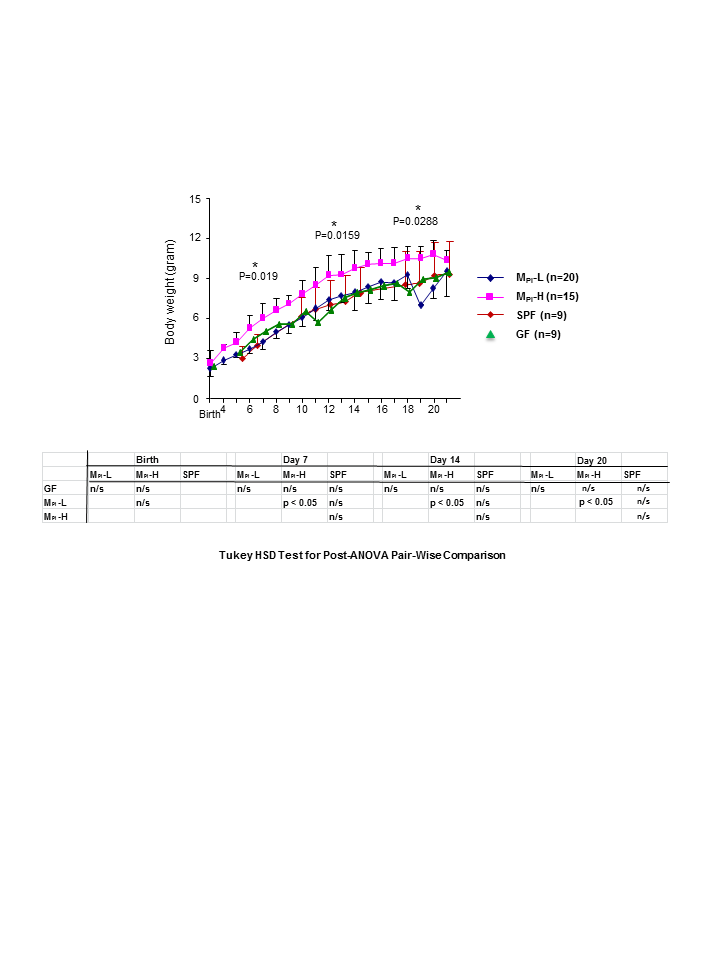

Supplement: S3 Fig — Growth curve of MPI-L (n = 20), MPI-H (n = 15), GF (n = 9) and SPF (n = 9) from birth to weaning. The daily weights are presented as mean (g) ± SEM. One-way ANOVA with post-hoc Tukey’s HSD test was used to compare the groups. (TIF) [file pone.0124504.s003.tif]
